# Immunotherapy Bridge 2021 and Melanoma Bridge 2021: meeting abstracts

**DOI:** 10.1186/s12967-022-03290-1

**Published:** 2022-04-05

**Authors:** 

## Oral Communications

### PIVOT-12: A phase 3 randomized study of adjuvant bempegaldesleukin (BEMPEG) plus nivolumab (NIVO) vs NIVO in completely resected cutaneous melanoma at high risk for recurrence

#### Alexander M M Eggermont^1,*^, Paolo A Ascierto^2^, Nikhil I Khushalani^3^, Dirk Schadendorf^4^, Genevieve M Boland^5^, Adi Diab^6^, Jeffrey S Weber^7^, Karl D Lewis^8^, Daniel Johnson^9^, Georgina V Long^10^, Sue Currie^11^, Mann Muhsin^12^, Mary Tagliaferri^12^, Matteo S Carlino^13^

##### ^1^Princess Máxima Center for Pediatric Oncology and the University Medical Center Utrecht, Utrecht, The Netherlands, ^2^Department of Melanoma, Cancer Immunotherapy and Development Therapeutics, Istituto Nazionale Tumori IRCCS Fondazione G. Pascale, Naples, Italy, ^3^Department of Cutaneous Oncology, H. Lee Moffitt Cancer Center, Tampa, FL, USA, ^4^Department of Dermatology, University Hospital Essen, Essen, Germany, ^5^Division of Surgical Oncology, Massachusetts General Hospital, Boston, MA, USA, ^6^Department of Melanoma Medical Oncology, The University of Texas MD Anderson Cancer Center, Houston, TX, USA, ^7^Department of Medical Oncology, Laura and Isaac Perlmutter Cancer Center at NYU Langone Health, New York, NY, USA, ^8^Division of Medical Oncology, Department of Medicine, University of Colorado Cancer Center, Aurora, CO, USA, ^9^ Department of Medical Oncology, Ochsner Medical Center, New Orleans, LA, USA, ^10^Melanoma Institute Australia, The University of Sydney, and Royal North Shore and Mater Hospitals, Sydney, Australia, ^11^formerly of Nektar Therapeutics, San Francisco, CA, USA,^12^Nektar Therapeutics, San Francisco, CA, USA, ^13^Department of Medical Oncology, Westmead and Blacktown Hospitals and Melanoma Institute Australia, The University of Sydney, Sydney, Australia

###### Correspondence: Alexander M.M. Eggermont (Email address: alexander.eggermont@prinsesmaximacentrum.nl)

*Journal of Translational Medicine* 2022; **20(1)**: 1

**Background:** Patients with stage III or IV melanoma are at high risk for recurrence following surgical resection. Nivolumab (NIVO), an immune checkpoint inhibitor (ICI), is a standard-of-care adjuvant therapy for patients with resected stage III/IV melanoma, with a 4-year recurrence-free survival (RFS) of 52% [1]. Advances in adjuvant therapy (e.g. use of novel combinations), have the potential to reduce or delay recurrence, and thus extend survival in patients with resected cutaneous melanoma.

Bempegaldesleukin (BEMPEG, NKTR-214) is an immunostimulatory interleukin-2 (IL-2) cytokine prodrug, engineered to deliver a controlled, sustained, and preferential IL-2 pathway signal. BEMPEG (monotherapy and in combination with NIVO) increases proliferation and infiltration of CD8^+^ cytotoxic T cells and natural killer cells with limited expansion of unwanted regulatory T cells in the tumor microenvironment [2–4]. Additionally, BEMPEG upregulates programmed cell death protein 1 (PD-1) on T cells as well as expression of PD-ligand 1 (PD-L1) in tumor tissue. This mechanism of action of BEMPEG supports its evaluation in combination with an ICI. In a phase 2 cohort of the PIVOT-02 trial (NCT02983045), BEMPEG plus NIVO was well tolerated and produced deep and durable responses in previously untreated patients with metastatic melanoma (N = 41) [4]. This phase 3, randomized, open-label study (PIVOT-12; NCT04410445) is exploring BEMPEG plus NIVO as adjuvant treatment for patients with completely resected cutaneous melanoma at high risk for recurrence.

**Patients and methods:** Eligible patients are age ≥ 12 years with histologically confirmed stage IIIA (lymph node metastases > 1 mm), IIIB/C/D, or IV (M1a/b/c/d) cutaneous melanoma (AJCC, 8th edition) that has been completely surgically resected (no evidence of residual disease) within 12 weeks prior to randomization (Fig. 1). Other eligibility criteria include tumor tissue provided for central analysis of PD-L1 expression (Dako PD-L1 IHC 28-8 pharmDx), no prior therapy for melanoma (except surgery and/or adjuvant radiation for CNS lesions), and no prior immunotherapy. Eligible patients will be randomized 1:1 to Arm A (BEMPEG 0.006 mg/kg IV Q3W + NIVO 360 mg IV Q3W) or Arm B (NIVO 480 mg IV Q4W). The primary objective is to compare the efficacy, as measured by RFS by BICR, of BEMPEG plus NIVO vs NIVO alone. Secondary endpoints include overall survival, distant metastasis-free survival (by BICR and investigator assessment in patients with stage III disease), safety and tolerability, and patient-reported outcomes. This trial is currently recruiting globally (Australia, Europe, New Zealand, and the United States), with a planned total enrollment of 950 patients.
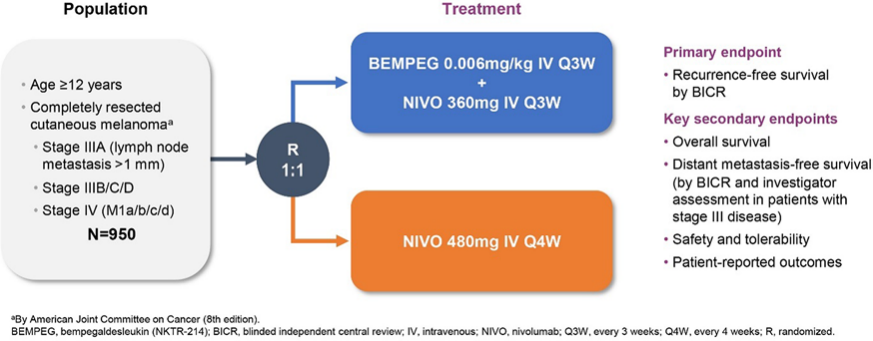


**Figure 1**. PIVOT-12 study design

**Trial registration:** ClinicalTrials.gov number NCT04410445.

## References


Ascierto PA, Del Vecchio M, Mandalá M et al. Adjuvant nivolumab versus ipilimumab in resected stage IIIB-C and stage IV melanoma (CheckMate 238): 4-year results from a multicentre, double-blind, randomised, controlled, phase 3 trial. Lancet Oncol. 2020;21:1465–77.Bentebibel S-E, Hurwitz ME, Bernatchez C et al. A first-in-human study and biomarker analysis of NKTR-214, a novel IL2rβγ-biased cytokine, in patients with advanced or metastatic solid tumors. Cancer Discov 2019;9:711–21.Diab A, Tannir NM, Bentebibel S-E et al. Bempegaldesleukin (NKTR-214) plus nivolumab in patients with advanced solid tumors: Phase 1 dose-escalation study of safety, efficacy and immune activation (PIVOT-02). Cancer Discov 2020;10:1158–73.Diab A, Tykodi SS, Daniels GA et al. Bempegaldesleukin plus nivolumab in first-line metastatic melanoma. J Clin Oncol 2021;26:2914–25.


### Harnessing therapy resistant tumor microenvironment with lipid nanoparticles carrying miR-199b-5p and miR-204-5p to potentiate MAPKi in BRAF-mutant metastatic melanoma

#### Luigi Fattore^1^, V. Campani^2^, E. Marra^3^, G. Cafaro^4^, D. Liguoro^5^, C.F. Ruggiero^6^, V. Castaldo^5^, L. Aurisicchio^3^, P.A. Ascierto^6^, G. De Rosa^2^, R. Mancini^5^, G. Ciliberto^7^

##### ^1^Department of Research, Advanced Diagnostics and Technological Innovation, Translational Research Area, IRCCS Regina Elena National Cancer Institute, Rome, Italy; ^2^ Department of Pharmacy, University of Naples Federico II, Naples, Italy; ^3^ Takis s.r.l., Rome, Italy; ^4^ Preclinical Models and New Therapeutic Agents Unit, IRCCS Regina Elena National Cancer Institute, Rome, Italy; ^5^ Department of Molecular and Clinical Medicine, University of Roma “Sapienza”, Rome, Italy; ^6^ Istituto Nazionale Tumori IRCCS, "Fondazione G. Pascale", Naples, Italy; ^7^ Scientific Directorate, IRCSS Regina Elena National Cancer Institute, Rome, Italy

###### Correspondence: Luigi Fattore (Email address: luigi.fattore@ifo.gov.it)

*Journal of Translational Medicine* 2022; **20(1)**: 2

**Background:** The efficacy of BRAF + MEK inhibitors for melanoma patients harboring BRAF mutations is limited by drug resistance; from here, the need to identify additional therapeutic approaches. Towards this goal, our group has demonstrated that microRNAs act as facilitators or antagonists of this phenomenon. This has both diagnostic and therapeutic implications; here, we have addressed the latters. Our nanotechnology approach, aimed to overcome the biopharmaceutical issues of the use of miRNAs in therapy*, i.e.* their rapid degradation in bloodstream and the poor intracellular uptake, has been to use lipid nanoparticles (LNPs) carrying the oncosuppressive miR-204-5p and miR-199b-5p for in vitro and in vivo efficacy studies.

**Material and Methods:** BRAF-mutant melanoma cell lines, namely M14 and A375, and their MAPKi-resistant counterparts have been subjected to: a) RNA-seq experiments and b) treatments using LNPs carrying miR-204-5p and miR-199b-5p. Gene Set Enrichment Analysis (GSEA) has been interrogated to identify the molecular pathways/genes affected by these miRNAs. Elisa assays have been used to assess the levels of cytokines in media coming from LNP-treated or not MAPKi-resistant cells. CD1 nude mice have been injected with M14 and A375 cells and treated with BRAFi + MEKi in the presence or not of LNPs to assess the efficacy of these combinatorial regimens.

**Results:** Starting from RNA-seq of two BRAF-mutant melanoma models rendered resistant to MAPKi in vitro implemented with GSEA bioinformatics analyses, we identified the molecular pathways/genes affected by oncosuppressive miR-204-5p and miR-199b-5p. They mostly rewire the alteration of cytokines and chemokines responsible for EMT, hypoxia and extracellular matrix degradation. In particular, we have demonstrated that VEGFa, TGFb1, CCL5 and CXCL2 are aberrantly released by drug resistant melanoma cells and their levels are restored upon LNP stimulation in vitro. According to the capability of these soluble factors to reprogram microenvironment responsible for tumor progression and therapy failure, we observed that cell media coming from resistant cells is able to trigger the migration and polarization of macrophages toward a pro-tumoral M2-type phenotype. Again, this phenomenon was strongly impaired by LNP treatments. Finally, using mouse models xenografted with M14 and A375 cells, we have demonstrated that LNPs carrying miR-204-5p and miR-199b-5p are able to reduce tumor growth as single agents and better when combined with MAPKi.

**Conclusions:** To our knowledge this is the first study combining LNPs carrying oncosuppressive miRNAs with target therapy in cancer and pave the way to propose them as a novel therapeutic option for BRAF-mutant melanoma patients.

### Nemvaleukin alfa (ALKS 4230) monotherapy in patients with advanced melanoma: ARTISTRY-1

#### Karl Lewis^1^, Valentina Boni^2,3^†, Emiliano Calvo^3^, Olivier Dumas^4^, David F. McDermott^5^, Sang Joon Shin^6^, Yan Wang^7^, Yangchun Du^7^, Lei Sun^7^, Monali Desai^7^, Carlos Mayo^7^, Julie R. Graham^7^*, Ira Winer^8^, Piotr Tomczak^9^

##### ^1^Department of Medicine, University of Colorado Denver Anschutz Medical Campus, Aurora, CO, USA; ^2^NEXT Oncology Madrid, Hospital Universitario Quirónsalud Madrid, Madrid, Spain; ^3^START Madrid-CIOCC, Centro Integral Oncológico Clara Campal, Madrid, Spain; ^4^CHU de Québec-Université Laval, Québec City, QC, Canada; ^5^Beth Israel Deaconess Medical Center, Boston, MA, USA; ^6^Yonsei Cancer Center, Yonsei University College of Medicine, Seoul, Korea; ^7^Alkermes, Inc., Waltham, MA, USA; ^8^Barbara Ann Karmanos Cancer Institute, Wayne State University, Detroit, MI, USA; ^9^Centrum Medyczne Pratia, Poznan, Poland; †At time of study

###### Correspondence: Juie R. Graham (Email address: Julie.Graham@Alkermes.com)

*Journal of Translational Medicine* 2022; **20(1)**: 3

**Background:** Despite recent melanoma treatment advances, mucosal melanoma remains particularly difficult to treat with response rates to frontline checkpoint inhibitor (CPI) monotherapy and progression-free survival times of < 10% and ~ 3 months, which are approximately half the figures for cutaneous melanoma. Responses are even worse in CPI-experienced patients. Nemvaleukin alfa (nemvaleukin) is a novel engineered cytokine that selectively binds to the intermediate-affinity IL-2 receptor, preferentially activating and expanding antitumor CD8^+^ T and NK cells, with minimal expansion of T_regs_. Nemvaleukin is being evaluated for treatment of advanced solid tumors, including melanoma, in ARTISTRY-1 (NCT02799095).

**Materials and methods:** Eligible patients with advanced melanoma (cutaneous, mucosal, uveal, acral) previously treated with a CPI were enrolled into a melanoma-specific cohort of ARTISTRY-1 Part B. Intravenous nemvaleukin (6 µg/kg) was administered for 5 days every cycle (14 days, cycle 1; 21 days, cycle 2 +). If patients had disease progression (after ≥ 2 cycles) or stable disease (after ≥ 4 cycles), they could enroll into Part C to receive nemvaleukin and pembrolizumab. Outcomes presented include antitumor activity (RECIST v1.1), pharmacodynamics, and safety as of August 2021.

**Results:** Forty-seven patients, all CPI-experienced, with advanced melanoma received nemvaleukin in Part B (≤ 28 cycles). Median age was 66 years (range, 37–80); median prior lines of therapy was 3 (range, 1–6). Of 46 evaluable patients, 33 had stable disease. Partial responses were observed in 4 patients, 2 (1 confirmed) of 6 with mucosal melanoma and 2 (1 confirmed) of 40 with cutaneous melanoma. Nemvaleukin induced robust expansion of CD8^+^ T and NK cells, with minimal effect on T_regs_. Treatment-emergent adverse events (AEs) (> 45%), regardless of causality, were pyrexia (66.0%) and nausea (51.1%). Grade ≥ 3 nemvaleukin-related AEs (> 10%) were neutropenia (40.4%) and decreased neutrophil count (17.0%). There were no deaths due to AEs. Three patients had AEs resulting in discontinuation: intestinal obstruction and failure to thrive (both unrelated to treatment) and confusion (related to treatment). Thirteen patients continue monotherapy (Part B) and 22 rolled over to Part C (combination therapy). Of 12 evaluable patients receiving combination therapy, 6 had stable disease, 3 of whom had progression on monotherapy. No additional safety signals were observed.

**Conclusions:** Nemvaleukin was well tolerated and provided evidence of antitumor activity in CPI-experienced patients with advanced melanoma. The US FDA granted nemvaleukin Orphan Drug and Fast Track designations for mucosal melanoma. A phase 2 study (ARTISTRY-6) is evaluating nemvaleukin in advanced mucosal or cutaneous melanoma.

**Trial Registration:** ClinicalTrials.gov NCT02799095.

**Funding: **This study is funded by Alkermes, Inc.

**Acknowledgments:** The authors would like to thank all the patients who are participating in this study and their families. The study is sponsored by Alkermes, Inc. Medical writing and editorial support was provided by Jennifer Klem, PhD, Parexel International, and funded by Alkermes, Inc.

### Clinical feasibility and treatment outcomes with unselected autologous tumor-infiltrating lymphocyte therapy in patients with advanced cutaneous melanoma

#### Robert E Hawkins,^1,2,3^* Yizhou Jiang,^1^ Paul C Lorigan,^2^ Fiona C Thistlethwaite,^2,3^ Manon Pillai,^2^ Martine Thomas,^1^ Natalia Kirillova,^1^ John S Bridgeman,^1^ Gray Kueberuwa,^1^ Ryan D Guest,^1^ Zachary J Roberts^1^

##### ^1^Instil Bio, Inc., Dallas, TX, USA; ^2^Department of Medical Oncology, The Christie, NHS Foundation Trust, Manchester, United Kingdom; ^3^Division of Cancer Sciences, University of Manchester, Manchester, United Kingdom

###### Correspondence: Robert Hawkins (Email address: Robert.Hawkins@instilbio.com)

*Journal of Translational Medicine* 2022; **20(1)**: 4

**Background**: Patients with advanced melanoma who relapse following immune checkpoint inhibition or targeted therapy have limited treatment options and poor outcomes. The intrinsic antitumor activity and broad T-cell receptor repertoire of unselected autologous tumor-infiltrating lymphocytes (TILs) may provide advantages over other treatments in solid tumors, including checkpoint inhibitor–refractory melanoma.

**Material and methods**: This is a retrospective analysis of a single-center experience of TILs for patients with advanced cutaneous melanoma and no standard of care treatment options. Unselected autologous TILs derived from digested tumors were manufactured under a Medicines and Healthcare Products Regulatory Agency Manufacturing Specials license. Patients received lymphodepleting chemotherapy (cyclophosphamide, fludarabine [Cy/Flu]), followed by TIL infusion and post-TIL high-dose IL-2 on a compassionate use basis. Efficacy was investigator assessed by CT/MRI for 15 patients per Response Evaluation Criteria in Solid Tumors (RECIST) version 1.1 (RECIST-evaluable group); 6 additional patients were followed using standard imaging techniques (e.g., CT, PET) and clinical monitoring but did not have quantitative tumor burden measurements (non-RECIST–evaluable group). Clinically significant adverse events (AEs) post-TIL infusion were reported. Data cutoff was December 31, 2019.

**Results:**: Between October 2011 and August 2019, 21 patients were treated with Cy/Flu, TILs, and high-dose IL-2. All patients had high-risk metastatic disease—33% had stage IV M1d, the median number of disease sites was 4, and patients received an average of 3 prior therapies (any checkpoint inhibitor, 91%; PD-1 inhibitor [PD-1i], 57%; BRAFi, 52%; MEKi, 24%). With a median follow-up of 52.2 months, the overall response rate in RECIST-evaluable patients (n = 15) was 53% (complete response rate, 13%); the disease control rate was 73%. Additional durable responses were observed in the non-RECIST–evaluable group (n = 6). Responses were generally consistent across subgroups, including age, number of disease sites, tumor burden, brain metastases, number of prior therapies, and prior PD-1i, BRAFi, and MEKi. For all treated patients, the median overall survival was 21.3 months. AEs were generally self-limited and consistent with Cy/Flu and high-dose IL-2. Common any-grade AEs (≥ 30% of patients) were thrombocytopenia (62%), pyrexia (57%), and rigors (43%); no treatment-related deaths were observed.

**Conclusions:** The high response rate observed in this series highlights the successful bench-to-bedside application of unselected autologous TILs to address unmet medical need in advanced melanoma. Use of tumor digests as starting material for manufacturing of TILs demonstrates the feasibility of this approach. A multicenter phase 2 trial of this therapy in advanced melanoma is currently enrolling (DELTA-1; NCT05050006).

### Phase 3 randomized trial comparing tebentafusp with investigator’s choice in first line metastatic uveal melanoma

#### Sophie Piperno-Neumann^1^, Jessica C. Hassel^2^, Piotr Rutkowski^3*^, Jean-Francois Baurain^4^, Marcus O. Butler^5^, Max Schlaak^6^, Ryan J. Sullivan^7^, Sebastian Ochsenreither^8^, Reinhard Dummer^9^, John M. Kirkwood^10^, Anthony, M. Joshua^11^, Joseph J. Sacco^12^, Alexander N. Shoushtari^13^, Marlana Orloff^14^, Richard D. Carvajal^15^, Omid Hamid^16^, Shaad E. Abdullah^17^, Chris Holland^17^, Howard Goodall^17^, Paul Nathan^18^

##### ^1^Institut Curie, Paris, France; ^2^University Hospital Heidelberg, Heidelberg, Germany; ^3^Sklodowska-Curie Memorial Cancer Center, Warsaw, Poland; ^4^Medical Oncology Department, King Albert II Cancer Institute, Cliniques Universitaires Saint-Luc, Brussels, Belgium; ^5^Princess Margaret Cancer Centre, Toronto, ON, Canada; ^6^Department of Dermatology and Allergy, University Hospital, Munich, Germany; ^7^Massachusetts General Hospital, Boston, MA, United States; ^8^Charité-Universitätsmedizin Berlin, Berlin, Germany; ^9^University Hospital of Zürich, Zurich, Switzerland; ^10^University of Pittsburgh Medical Center, Pittsburgh, PA, United States; ^11^Saint Vincent's Hospital, Sydney, Australia; ^12^The Clatterbridge Cancer Centre, Wirral, United Kingdom; ^13^Memorial Sloan-Kettering Cancer Center, New York, NY, United States; ^14^Thomas Jefferson University Hospitals, Philadelphia, PA, United States; ^15^Herbert Irving Comprehensive Cancer Center, Columbia University Irving Medical Center, New York, NY, United States; ^16^The Angeles Clinic and Research Institute, A Cedars Sinai Affiliate, Los Angeles, CA, United States; ^17^Immunocore Ltd, Abingdon, United Kingdom; ^18^Mount Vernon Cancer Centre, Northwood, United Kingdom

###### Correspondence: Piotr Rutkowski (Email address: Piotr.Rutkowski@pib-nio.pl)

*Journal of Translational Medicine* 2022; **20(1)**: 5

**Background:** Metastatic uveal melanoma (mUM) has a poor prognosis with a 1-yr OS rate of 52%. No systemic treatment has proven an OS benefit in randomized trials. Tebentafusp (tebe), a bispecific consisting of an affinity-enhanced T cell receptor fused to an anti-CD3 effector that can redirect T cells to target gp100 + cells, has shown promising activity in previously treated mUM pts. Here, we report the primary analysis of overall survival (OS) in the intention-to-treat population (ITT) of a Ph3 trial of tebe vs. investigator’s choice (IC) as first line (1L) therapy in pts with mUM [NCT03070392].

**Material and methods:** In this randomized, open-label, Ph3 trial, 1L HLA-A*02:01 + pts with mUM were randomized 2:1 to receive tebe or IC of pembrolizumab, ipilimumab or dacarbazine, stratified by LDH. The primary endpoint was OS, defined as the time from randomization to death from any cause. Dual primary objectives were to evaluate 1) OS in the ITT population by comparing all tebe-randomized pts to all IC-randomized pts; and 2) OS in tebentafusp-treated patients with rash during week 1 versus all IC-treated patients. Secondary endpoints included safety and RECIST-defined overall response rate, progression free survival and disease control rate. Here we present the OS in the ITT population. The study was unblinded by an independent data monitoring committee at the first pre-specified interim analysis. This analysis was conducted on the first interim analysis (data extracted Nov 2020).

**Results:** 378 pts were randomized to tebe (252) or IC, including pembrolizumab (103), ipilimumab (15) or dacarbazine (7). Tebe significantly prolonged OS compared to IC (HR 0.51; 95% CI 0.36–0.71; P < 0.0001) in the ITT population, with estimated 1-yr OS rate of 73.2% (95% CI 66.3–78.9) vs 57.5% (95% CI 47.0–66.6), respectively.

Most common TRAEs were skin-related (gp100+ melanocytes) or cytokine-mediated (T cell activation) and included pyrexia, pruritus, and rash. These AEs decreased in frequency and severity after initial 3-4 doses and were generally manageable with standard interventions. In the tebe arm, the rate of treatment discontinuation due to TRAEs was low (<4%), and there were no treatment-related deaths.

**Conclusions:** In 1L treatment of mUM pts, tebe monotherapy significantly improved OS compared to IC; the first investigational therapy to improve OS in pts with mUM. Tebe had a predictable and manageable AE profile with a low rate of related discontinuation. Tebe is the first TCR therapeutic to demonstrate an OS benefit.

**Trial registration:** NCT03070392.

©AACR. Original forum for presentation; Piperno-Neumann S, Hassel JC, Rutkowski P, et al. Abstract CT002: Phase 3 randomized trial comparing tebentafusp with investigator's choice in first line metastatic uveal melanoma. Cancer Res July 1 2021 (81) (13 Supplement) CT002; https://doi.org/10.1158/1538-7445.AM2021-CT002

### Mechanistic insights into the role of B cells in radioiodine therapy of differentiated thyroid cancer associated with type 2 diabetes mellitus

#### Adina E Stanciu^1,*^, Madalina Bolovan^1^, Anca Zamfirescu^2^, Marcel M Stanciu^3^, Marieta E Panait^4^

##### ^1^Department of Carcinogenesis and Molecular Biology, Institute of Oncology” Prof.Dr.Alex.Trestioreanu” Bucharest, Romania; ^2^Department of Radionuclide Terapy, Institute of Oncology” Prof.Dr.Alex.Trestioreanu” Bucharest, Romania; ^3^University Politehnica of Bucharest, omania; ^4^Department of Cancer Biology, Institute of Oncology” Prof. Dr. Alex.Trestioreanu” Bucharest, Romania

###### Correspondence: Adina E. Stanciu (Email address: adinaelenastanciu@yahoo.com)

*Journal of Translational Medicine* 2022; **20(1)**: 6

**Background:** The prevalence of type 2 diabetes mellitus (T2DM) is significantly increased in women with differentiated thyroid cancer (DTC) and influences overall survival. T2DM is characterized by a progressive status of chronic, low-grade inflammation with an altered number and function of immune cells, both innate and acquired immunity [1]. Tumor necrosis factor receptor 2 (TNFR2) expression is linked to tolerogenic immune reactions and cells with suppressor function, including a subset of T-regulatory cells [2]. Ablative radiotherapy dramatically increases T-cell priming in draining lymphoid tissues, leading to reduction/eradication of the primary tumor or distant metastasis in a CD8 + T cell-dependent fashion [3]. We hypothesized that B cells play a key role in controlling the T-cell responses to radioiodine (131I) therapy in patients with DTC and DTC associated with T2DM (DTC + T2DM). Our study aimed to evaluate the effects of 131I on TNFR2 and B cells in DTC and DTC + T2DM patients.

**Material and Methods:** Peripheral blood was collected from 42 female patients with DTC (mean age 44.6 ± 10.7 years) and 16 with DTC + T2DM (mean age 50.1 ± 9.8 years) before and 4 days after the I-131 administration (3.7 GBq). The distribution of circulating lymphocyte subpopulations was measured by flow cytometry and the serum levels of TNFR2 by ELISA. A dose calibrator was used to measure blood activity with a microcurie accuracy.

**Results:** Radioactivity of the blood samples was higher in DTC + T2DM patients than in those without T2DM (*P* < 0.001). Increased radioactivity of blood collected 4 days after the same dose of ^131^I /patient intake indicates a low ^131^I uptake in the DTC + T2DM group. In the presence of T2DM, ^131^I led to an increase in the serum TNFR2 concentration (P = 0.01), CD19 + B-lymphocytes (P = 0.03), and a reduction in CD8 + T-cells count number (P = 0.02). In contrast, in DTC patients, ^131^I therapy resulted in enhanced anti-tumor immunity mediated by CD8 + T-cells (P = 0.04) by inhibiting TNFR2.

**Conclusions:** The therapeutic efficacy of targeted radionuclide therapy with high-dose depends on the presence of CD8 + T cells both before and after ^131^I intake. Our results suggest that proliferation of CD8 + T cells after ^131^I is reduced in the presence of TNFR2 and CD-19 + B cells in DTC + T2DM.

**Funding Acknowledgement: **This work was supported by a grant from the Romanian Ministry of Education and Research, CCCDI—UEFISCDI, project number PN-III-P2-2.1-PED-2019–3313, within PNCDI III.

## References


de Candia P, Prattichizzo F, Garavelli S, De Rosa V, Galgani M, Di Rella F, Spagnuolo MI, Colamatteo A, Fusco C, Micillo T, Bruzzaniti S, Ceriello A, Puca AA, Matarese G. Type 2 Diabetes: How Much of an Autoimmune Disease? Front Endocrinol (Lausanne). 2019; 10:451. https://doi.org/10.3389/fendo.2019.00451.Stanciu AE. Cytokines in heart failure. Adv Clin Chem. 2019; 93:63–113. https://doi.org/10.1016/bs.acc.2019.07.002Gheorghe DC, Stanciu MM, Zamfirescu A, Stanciu AE**.** TNF-α May Exert Different Antitumor Effects in Response to Radioactive Iodine Therapy in Papillary Thyroid Cancer with/without Autoimmune Thyroiditis. Cancers (Basel). 2021; 13(14):3609. https://doi.org/10.3390/cancers13143609.


### Co-primary endpoint of overall survival for tebentafusp (tebe)-induced rash in a Phase 3 randomized trial comparing tebe vs. investigator’s choice (IC) in first line metastatic uveal melanoma

#### Jessica C. Hassel^1^, Piotr Rutkowski^2^, Jean-Francois Baurain^3^, Marcus O. Butler^4^, Max Schlaak^5^, Ryan J. Sullivan^6*^, Sebastian Ochsenreither^7^, Reinhard Dummer^8^, John M. Kirkwood^9^, Anthony M. Joshua^10^, Joseph J. Sacco^11^, Alexander N. Shoushtari^12^, Marlana Orloff^13^, Richard D. Carvajal^14^, Omid Hamid^15^, Shaad E. Abdullah^16^, Chris Holland^16^, Howard Goodall^16^, Paul Nathan^17^, Sophie Piperno-Neumann^18^

##### ^1^University Hospital Heidelberg, Heidelberg, Germany; ^2^Sklodowska-Curie Memorial Cancer Center, Warsaw, Poland; ^3^Medical Oncology Department, King Albert II Cancer Institute, Cliniques Universitaires Saint-Luc, Brussels, Belgium; ^4^Princess Margaret Cancer Centre, Toronto, ON, Canada; ^5^Department of Dermatology and Allergy, University Hospital, Munich, Germany; ^6^Massachusetts General Hospital, Boston, MA, United States; ^7^Charité-Universitätsmedizin Berlin, Berlin, Germany; ^8^University Hospital of Zürich, Zurich, Switzerland; ^9^University of Pittsburgh Medical Center, Pittsburgh, PA, United States; ^10^Saint Vincent's Hospital, Sydney, Australia; ^11^The Clatterbridge Cancer Centre, Wirral, United Kingdom; ^12^Memorial Sloan-Kettering Cancer Center, New York, NY, United States; ^13^Thomas Jefferson University Hospitals, Philadelphia, PA, United States; ^14^Herbert Irving Comprehensive Cancer Center, Columbia University Irving Medical Center, New York, NY, United States; ^15^The Angeles Clinic and Research Institute, A Cedars Sinai Affiliate, Los Angeles, CA, United States; ^16^Immunocore Ltd, Abingdon, United Kingdom; ^17^Mount Vernon Cancer Centre, Northwood, United Kingdom; ^18^Institut Curie, Paris, France

###### Correspondence: Ryan J. Sullivan (Email address: rsullivan7@partners.org)

*Journal of Translational Medicine* 2022; **20(1)**: 7

**Background:** Tebe is a bispecific consisting of an affinity-enhanced T cell receptor fused to an anti-CD3 effector that can redirect T cells to target gp100 + cells. In this Phase (Ph) 3, randomized trial of first line (1L) metastatic uveal melanoma (mUM) [NCT03070392], tebe significantly improved overall survival (OS) vs. investigator’s choice (IC) in the intention-to-treat population (ITT). In previous trials, tebe-related skin adverse events (AEs), hypothesized to be on-target, off-tumor activity against gp100-expressing melanocytes, were associated with improved OS. This association was tested prospectively as co-primary endpoint in the Ph3 study.

**Material and methods:** 378 1L HLA-A*02:01 + mUM pts were randomized 2:1 to tebe (n = 252) or IC (n = 126). Co-primary endpoints were 1) OS in all randomized pts (ITT) and 2) OS in tebe-randomized pts who develop any grade rash in week 1 vs. all receiving IC. Rash was a composite of preferred AE terms. Melanocyte-related AEs (MRAEs) were defined as pigment change AEs in skin or hair. Overall study-wide alpha was controlled at 0.05, with 90% assigned to ITT and 10% to rash. This analysis was conducted on the first interim analysis (Nov-2020).

**Results:** In 245 tebe-treated pts, the most frequent skin-related AEs included rash (at any time) in 201 pts (82%), pruritis in 167 pts (68%), MRAEs in 109 pts (45%) and erythema in 69 pts (28%). While rash, erythema and pruritis mostly occurred in the first 4 weeks, MRAEs occurred after a median of 2.7 mo. Rash occurred in 146 pts (60%) by week 1; 179 pts (73%) by week 2; and 195 pts (80%) by week 3.

Tebe pts with week 1 rash had significantly longer OS vs. IC arm, HR 0.35 (95% CI 0.23, 0.53), p < 0.0001. The estimated 1-yr OS rates were 83% vs 58%, respectively. When expanded to include tebe pts with rash through week 3, the 1-yr OS rate of 75% was still numerically higher than IC. The 50 (20%) tebe pts who did not experience rash by week 3 had 1-yr OS rate of 55%.

**Conclusions:** In 1L mUM pts, tebe significantly improved OS compared to IC in the ITT analysis. Week 1 rash was associated with a very strong OS benefit. Therefore, rash may be a marker that the immune system can be mobilized by tebe to target gp100 + cells. The vast majority of tebe pts will develop a rash at some point, and tebe pts without rash may still derive benefit.

**Trial registration:** NCT03070392.

© 2021 American Society of Clinical Oncology, Inc. Reused with permission. This abstract was accepted and previously presented at the 2021 ASCO Annual Meeting. All rights reserved. Hassel JC, Rutkowski P, Baurain JF, et al. Co-primary endpoint of overall survival for tebentafusp (tebe)-induced rash in a phase 3 randomized trial comparing tebe versus investigator’s choice (IC) in first-line metastatic uveal melanoma. Journal of Clinical Oncology 39, no. 15_suppl (May 20, 2021) 9527–9527; https://doi.org/10.1200/JCO.2021.39.15_suppl.9527

### RATIONALE 302: Randomized, Phase 3 study of tislelizumab versus chemotherapy as second-line treatment for advanced unresectable/metastatic esophageal squamous cell carcinoma

#### Eric Van Cutsem,^1*^ Ken Kato,^2^ Sung-Bae Kim,^3^ Jaffer Ajani,^4^ Kuaile Zhao,^5^ Zhiyong He,^6^ Xinmin Yu,^7^ Yonqian Shu,^8^ Qi Luo,^9^ Jufeng Wang,^10^ Zhendong Chen,^11^ Zuoxing Niu,^12^ Jong-Mu Sun,^13^ Chen-Yuan Lin,^14^ Hiroki Hara,^15^ Roberto Pazo-Cid,^16^ Christophe Borg,^17^ Liyun Li,^18^ Aiyang Tao,^18^ Lin Shen^19^

##### ^1^University Hospitals Gasthuisberg Leuven and KULeuven, Leuven, Belgium; ^2^National Cancer Center Hospital, Tokyo, Japan; ^3^Asan Medical Center, University of Ulsan College of Medicine, Seoul, South Korea; ^4^University of Texas MD Anderson Cancer Center, Houston, Texas; ^5^Fudan Cancer Hospital, Shanghai, China; ^6^Fujian Cancer Hospital, Fujian Medical University Cancer Hospital, Fujian, China; ^7^Zhejiang Cancer Hospital, Hangzhou, China; ^8^Jiangsu Province Hospital, Jiangsu, China; ^9^The First Affiliated Hospital of Xiamen University, Fujian, China; ^10^The Affiliated Cancer Hospital of Zhengzhou University, Henan Cancer Hospital, Zhengzhou, China; ^11^2nd Hospital of Anhui Medical University, Anhui, China; ^12^Department of Medical Oncology, Shandong Cancer Hospital, Shandong Academy of Medical Sciences, Jinan, China; ^13^Samsung Medical Center, Sungkyunkwan University School of Medicine, Seoul, South Korea; ^14^China Medical University Hospital, and China Medical University, Taichung, Taiwan; ^15^Saitama Cancer Center, Saitama, Japan; ^16^Hospital Universitario Miguel Servet, Zaragoza, Spain; BeiGene Ltd, Beijing, China; ^17^Medical Oncology Department, University Hospital of Besançon, Besançon, France; ^18^BeiGene Ltd, Zhongguancun Life Science Park, Beijing, China; ^19^Department of Gastrointestinal Oncology, Key Laboratory of Carcinogenesis and Translational Research (Ministry of Education/Beijing), Peking University Cancer Hospital & Institute, Beijing, China

###### Correspondence: Eric Van Cutsem (Email address: eric.vancutsem@uzleuven.be)

*Journal of Translational Medicine* 2022; **20(1)**: 8

**Background:** Tislelizumab, alone and with chemotherapy, has demonstrated antitumor activity in patients with solid tumors including esophageal squamous cell carcinoma (ESCC; NCT03469557, NCT04068519).

**Material and methods:** This global Phase 3 study (NCT03430843) enrolled adults with histologically confirmed advanced or metastatic ESCC who progressed following prior systemic therapy. Eligible patients were randomized (1:1) to receive tislelizumab 200 mg intravenously every 3 weeks or investigator-chosen standard chemotherapy (ICC; paclitaxel, docetaxel, or irinotecan) and treated until disease progression, unacceptable toxicity, or withdrawal. Stratification factors included ICC option, region, and ECOG PS. The primary endpoint was overall survival (OS) in the intention-to-treat population. The key secondary endpoint was OS among programmed death-ligand 1–positive (PD-L1 +) patients (visually-estimated combined positive score ≥ 10% by VENTANA PD-L1 SP263 assay). Other secondary endpoints included (by RECIST v1.1) progression-free survival, overall response rate (ORR), duration of response (DoR), and safety.

**Results:** Overall, 512 patients (median age: 62 years; range 35–86 years) from 132 sites in 10 countries in Asia (n = 404; 79%), Europe, and North America (n = 108; 21%) were randomized to tislelizumab (n = 256) or ICC (n = 256). Of these, 157 patients (tislelizumab, n = 89; ICC, n = 68) were PD-L1 + . As of December 1, 2020, median study follow-up was 8.5 months with tislelizumab arm and 5.8 months with ICC arm. The study met its primary endpoint: tislelizumab significantly improved OS vs ICC (median OS: 8.6 vs 6.3 months; HR 0.70, 95% CI: 0.57–0.85, *P* = 0.0001). Significant OS improvement was also seen with tislelizumab in the PD-L1 + population (median OS: 10.3 vs 6.8 months; HR 0.54, 95% CI: 0.36–0.79, *P* = 0.0006). Survival benefit was consistently observed across predefined subgroups, including baseline PD-L1 status and region. Tislelizumab was associated with higher ORR (20.3% vs 9.8%) and a more durable response (median DoR: 7.1 vs 4.0 months; HR 0.42, 95% CI: 0.23–0.75) than ICC. Fewer patients had Grade ≥ 3 treatment-emergent adverse events (AEs) (46% vs 68%) and Grade ≥ 3 treatment-related TEAEs (19% vs 56%) with tislelizumab vs ICC. Fewer patients discontinued tislelizumab versus ICC (7% vs 14%) due to a treatment-related TEAE.

**Conclusions:** Tislelizumab demonstrated statistically significant and clinically meaningful improvement in OS versus ICC in patients with advanced or metastatic ESCC who had disease progression during or after first-line systemic therapy. Tislelizumab showed a higher and longer response and had a more favorable safety profile compared with ICC.

**Funding/Acknowledgement Statement:** This study was sponsored by BeiGene, Ltd. Editorial assistance was provided by Arpita Kulshrestha, PhD and Cheryl Casterline, MA (Peloton Advantage, LLC, an OPEN Health company, Parsippany, NJ), and funded by the study sponsor.

### Preliminary results from the skin cancer cohorts from an ongoing multi-cohort phase 2 clinical trial of RP1 combined with nivolumab (IGNYTE)

#### Ari M Vanderwalde^1*^, Mohammed M Milhem^2^, Tawnya L Bowles^3^ Joseph J Sacco^4^, Jiaxin Niu^5^, Katy K Tsai^6^ Jason A Chesney^7^, Bartosz Chmielowski^8^, Adel Samson^9^, Terence D Rhodes^10^ Praveen K Bommareddy^12^, Lavita Menezes^12^, Syed Raza^12^, Shui He^12^, Robert S Coffin^12^, Kevin Harrington^13^ Mark R Middleton^14^

##### ^1^West Cancer Center and Research Institute, Germantown, TN, USA; ^2^Holden Comprehensive Cancer Center, University of Iowa, Iowa City, IA, USA; ^3^Intermountain Med Ctr, Murray, UT, USA; ^4^Clatterbridge Cancer Centre, Wirral, United Kingdom and University of Liverpool, Liverpool, UK; ^5^Banner MD Anderson Cancer Center, Gilbert, AZ, USA; ^6^Helen Diller Family Comprehensive Cancer Center Cutaneous Oncology San Francisco, CA, USA; ^7^James Graham Brown Cancer Center, University of Louisville, Louisville, KY, USA; ^8^ University of California Los Angeles, Los Angeles, CA, USA; ^9^University of Leeds, Leeds, UK; ^10^ Intermountain Med Ctr, St George, UT, USA; ^12^Replimune Inc, Woburn, MA, USA; ^13^Royal Marsden NHS Foundation Trust, The Institute of Cancer Research, London, UK; ^14^Churchill Hospital, University of Oxford, Oxford, UK

###### Correspondence: Ari M Vanderwalde (Email address: avanderw@westclinic.com)

*Journal of Translational Medicine* 2022; **20(1)**: 9

**Background:** RP1 is an enhanced potency oncolytic version of HSV1 that expresses human GM-CSF and the fusogenic protein GALV-GP R- [1]. Preliminary data from the phase 1/2 trial demonstrated tolerability and anti-tumor activity for RP1 + nivolumab (nivo) [2]. Here, we present updated results from the melanoma and non-melanoma skin cancers (NMSC) cohorts from the trial.

**Material and Methods:** RP1 is administered via intratumoral injection Q2W, ≤ 10 mL/visit, first alone at a dose of 10^6^ PFU/mL and then starting with the 2^nd^ dose at 10^7^ PFU/mL in combination with nivo (240 mg IV Q2W for 4 mos then 480 mg IV Q4W up to 2 yrs) for up to 8 doses, with the option to re-initiate RP-1 if protocol specified criteria are met. Eligible patients (pts) must have at least one measurable & injectable tumor of ≥ 1 cm, ECOG 0–1, and no prior oncolytic therapy.

**Results:** As of June 2021, the combination continued to be generally well tolerated with no new safety signals identified. The objective response rate (ORR) in PD1 naïve cutaneous melanoma pts (n = 8) was 62.5% and 31.3% in anti-PD1 failed (n = 16). The ORR in CSCC (n = 15) was 60% including 46.6% durable CRs. ORR in BCC (n = 4), MCC (n = 4) and angiosarcoma (n = 5) were 25%, 75% and 60% respectively. Responses have been observed to be durable and to deepen over time.

**Conclusions:** RP1 in combination with nivo provides durable anti-tumor activity in pts with skin cancers, including CSCC, and anti-PD1/anti-CTLA-4 failed melanoma. Based on this data, the clinical trial has been expanded to include a registration directed cohort of pts who have anti-PD1 failed cutaneous melanoma (125 pts) and pts with anti-PD1 failed non-small cell lung cancer, anti-PD1 failed MSI-H cancers and anti-PD1 failed NMSC.

**Clinical Trial registration:** NCT03767348.

## References


Thomas S, Kuncheria L, Roulstone V, Kyula JN, Mansfield D, Bommareddy PK, Smith H, Kaufman HL, Harrington KJ, Coffin RS. Development of a new fusion-enhanced oncolytic immunotherapy platform based on herpes simplex virus type 1. J Immunother Cancer. 2019;7(1):214.Middleton M, Aroldi F, Sacco J, Milhem M, Curti B, VanderWalde A, Baum S, Samson A, Pavlick A, Chesney J, Niu J, Rhodes T, Bowles T, Conry R, Olsson-Brown A, Earl Laux D, Kaufman H, Bommareddy P, Deterding A, Samakoglu S, Coffin R, Harrington K. 422 An open-label, multicenter, phase 1/2 clinical trial of RP1, an enhanced potency oncolytic HSV, combined with nivolumab: updated results from the skin cancer cohorts. J Immunother Cancer. 2020; 8 (3).


### Neoadjuvant Ipilimumab/Nivolumab combination in locally advanced or oligometastatic melanoma

#### Pier Francesco Ferrucci^1,*^, Luisa Lanfrancone^1^, Luca Mazzarella^1^, Luigi Nezi^1^, Bruno Achutti Duso^1^, Teresa Manzo^1^, Fiorenza Lotti^1^, Sara Gandini^1^, Gianmarco Orsolini^1^, Elisabetta Pennacchioli^1^, Patrizia Gnagnarella^1^, Maria Teresa Fierro^2^, Rebecca Senetta^2^, Concetta Riviello^1^, Virginia Caliendo^2^, Pietro Quaglino^2^, Giovanni Mazzarol^1^, Giuseppina Bonizzi^1^, Emilia Cocorocchio^1^

##### ^1^Istituto Europeo di Oncologia IRCCS, Milano, Italy; ^2^ Università di Torino, Dermatologic Clinic, Torino, Italy

###### Correspondence: Pier Francesco Ferrucci (Email address: pier.ferrucci@ieo.it)

*Journal of Translational Medicine* 2022; **20(1)**: 10.

**Background:** We investigated the role of neoadjuvant combination immunotherapy, followed by surgery and adjuvant immunotherapy for locally advanced or oligometastatic melanoma patients (pts), within an open label, single arm and two sites study (European Institute of Oncology in Milan and University of Turin).

**Material and Methods:** Treatment schedule consisted in four primary cycles of inverted dose Ipilimumab 1 mg/kg and Nivolumab 3 mg/kg every 3 weeks, followed by radical surgery and adjuvant Nivolumab 480 mg every 4 weeks for 6 cycles. Primary objective was pathological complete remission (pCR) rate, according to International Neoadjuvant Melanoma Consortium (INMC) criteria, while secondary objectives were: safety, feasibility and efficacy; QoL; identification of molecular and immunological biomarkers of response and resistance; degree of immune activation; evaluation of the gut microbioma.

From March 2019 to April 2021, 43 pts were enrolled in the trial and, with an intent to treat of 35 pts, 34 completed the primary phase, 31 surgery and 26 the adjuvant phase (plus 2 pts who are still on-treatment). Four pts were withdrawn during primary phase for progression (2), toxicity (1) and consent withdrawal (1).

**Results:** Of the 31 pts who underwent surgery, 20 reached a pCR/near pCR (65%), while a pathological partial remission (pPR) was obtained in 4 (13%) and pathological no response (pNR) in 7 (22%) pts.

With a median follow-up of 17 months, 33/35 pts are alive. Treatment failure occurred in 9 pts: 2 pts progressed during primary phase and did not undergo surgery; 7 pts progressed during adjuvant (3 pts) or follow-up phase (4 pts). Six out of these 7 pts were classified as pNR at surgery, while the other, classified as pCR, did not receive adjuvant therapy. Both pts in stage IV relapsed, while 2 died as consequence of melanoma progression, and one for ischemic stroke after 5 months from adjuvant therapy while on CR.

Treatment related toxicities were mainly G1-2 and only 6 pts (17%) developed G3-4 adverse events (AE): 3 transaminitis, 1 pneumonitis, 1 myocarditis, 1 CPK increase and 1 dermatomiositis; all of them but two underwent to surgery after toxicity resolution.

Translational studies are ongoing on samples collected before and during therapy: whole exome sequencing and gut microbiota dynamics on longitudinal samples appear to be intriguing, showing some relationships with responses.

**Conclusions:** In conclusion, primary immunotherapy with Ipilimumab/Nivolumab is a feasible therapeutic option, able to achieve a high pCR/near pCR rate in pts affected by locally advanced/oligometastatic melanoma (primary objective met). Toxicity was lower than previously reported. Translational data evaluated longitudinally on each patient will be available at the time of the meeting and presented.

